# Estimation Strategy Selection Is Modulated by Snapshot Emotional Priming, but Not Math Anxiety

**DOI:** 10.3390/ijerph191610268

**Published:** 2022-08-18

**Authors:** Chuanlin Zhu, Xinyi Zhao, Xinhua Han, Yun Wang, Dianzhi Liu, Wenbo Luo

**Affiliations:** 1 School of Educational Science, Yangzhou University, Yangzhou 225002, China; 2 School of Foreign Languages, Suzhou University of Science and Technology, Suzhou 215009, China; 3 School of Education, Soochow University, Suzhou 215123, China; 4 Research Center of Brain and Cognitive Neuroscience, Liaoning Normal University, Dalian 116029, China; 5 Key Laboratory of Brain and Cognitive Neuroscience, Dalian 116029, China

**Keywords:** emotional priming, estimation strategy selection, math anxiety

## Abstract

The present study explored the role of snapshot emotional priming and math anxiety in estimation strategy selection. Participants were asked to complete a two-digit multiplication estimation task (e.g., 34 × 67) under explicit (Experiment 1) and implicit (Experiment 2) snapshot emotional priming conditions by freely choosing to use DU (down-up, e.g., doing 30 × 70 = 2100 for 34 × 67) or UD (up-down, e.g., doing 40 × 60 = 2400 for 34 × 67) strategies to arrive as close as possible to the correct answer. In Experiment 1, individuals’ estimation performance was positively influenced by explicit happy priming (shorter RT (reaction time)), while not affected by explicit fear priming. In Experiment 2, individuals’ estimation ACC (accuracy) when using the UD strategy was negatively affected by both implicit happy and fear priming, but their RT when using DU and UD strategies was positively impacted by implicit happy priming. In both experiments, the correlations between math anxiety and estimation performance (ACC, RT, and strategy selection adaptivity) was not significant. The present study suggests that fear priming was not always detrimental to individuals’ estimation performance, and happy priming did not always universally improve individuals’ estimation performance. Additionally, estimation strategy selection was not influenced by math anxiety.

## 1. Introduction

In daily life, we often encounter problems that are inconvenient to solve by mental arithmetic. For example, the price of potatoes is 2.99¥ per kilogram, so how much does it cost to buy 3 kg? In this case, we like to adopt estimation. Estimation refers to performing mental arithmetic adopting specified procedures or rules, thus arriving as close as possible to the correct answer [1]. Commonly used estimation strategies includes rounding down (RD) (i.e., do 40 × 70 for 42 × 73), rounding up (RU) (i.e., do 50 × 80 for 42 × 73), up-down (UD) (i.e., do 50 × 70 for 42 × 73), and down-up (DU) (i.e., do 40 × 80 for 42 × 73) [1,2]. When encountering different situations, people can choose different strategies to complete the estimation task and then achieve their expected goal, which is the estimation strategy selection.

Researchers have carried out in-depth research on estimation strategy selection, and they found that the performance efficiency of estimation strategy selection is influenced by multiple factors, such as age [3,4,5], effects of prior-task failure/success [6,7], mathematics achievement [8], stereotype [9], working memory [10,11], and emotions [12,13] such as math anxiety. In particular, numerous studies have looked at the relationship between math anxiety and math performance and demonstrated a negative correlation between the two [14,15,16]. For example, although not prominent in adults, Sun et al. [5] found that the strategy selection adaptivity of low math anxiety children was better than their high-anxiety counterparts. To be more specific, the two groups’ RT showed no significant difference, but the low (vs. high) math anxiety children’s ACC was higher. Additionally, Si et al. have explored the influence of math anxieties on estimation strategy selection [17]. In this study [17], participants were divided into two groups (high math anxiety group and low math anxiety group). They were asked to finish the two-digit addition estimation task by freely choosing which strategy to use. The behavioral results showed that the two groups’ ACC and RT for completing the estimation task showed no significant difference. However, the ERP (event-related potential) results showed that the N100 and N400 amplitudes of the high (vs. low) math anxiety groups were larger; meanwhile, the N400 latency of the high (vs. low) math anxiety groups was shorter. In total, these studies showed that individuals’ strategy selection adaptivity is related to their emotional experience (e.g., math anxiety).

In addition to anxiety, we experience a variety of emotions in our daily life, such as happiness, anger, fear, and so on. Previous studies have shown that individuals’ estimation performance was negatively influenced by negative snapshot emotional priming and positively influenced by positive snapshot emotional priming when they were required to complete estimation tasks by using the specified strategy (estimation strategy execution) [18,19]. Additionally, individuals’ estimation performance was also influenced by snapshot emotional priming style (explicit priming vs. implicit priming). For example, explicit fear priming reduced individuals’ RT. In contrast, implicit fear priming contributed to reducing individuals’ RT, while both explicit and implicit happy priming contributed to improving individuals’ estimation performance [2,19]. In summary, these studies indicated that individuals’ performance of estimation strategy execution was influenced by the snapshot emotional priming stimuli’s valence and the snapshot emotional priming style. Would estimation strategy selection be influenced by snapshot emotional priming? To our knowledge, no study has explored the role of snapshot emotional priming and math anxiety during estimation strategy selection.

To address this question, two 3 (priming emotion types: fear, happy, and neutral) × 2 (estimation strategy types: DU, UD) experimental designs were adopted in the present study. In Experiment 1, participants were required to complete a two-digit multiplication estimation task (e.g., 23 × 67) under explicit snapshot emotional priming conditions (which was achieved through the emotion judgment task), using DU or UD strategy. Experiment 2 was the same as Experiment 1, except that explicit snapshot emotional priming was replaced with implicit snapshot emotional priming (achieved through the gender judgment task). Since no studies have examined the influence of snapshot emotional priming on estimation strategy selection, we would formulate hypotheses in conjunction with research on the effect of snapshot emotional priming on estimation strategy execution.

The broaden-and-build theory of positive emotions proposed that positive emotional experiences contribute to improving cognitive flexibility [20,21], based on which we hypothesized that happy (vs. neutral and fear) priming would be beneficial to improving the individual’s estimation strategy selection adaptivity. Considering previous studies showing that RT was a more sensitive indicator than ACC when investigating individuals’ estimation performance [2,19,22,23], we hypothesized that the positive effect of happy priming mainly is manifested in shorter RTs (hypothesis one). Additionally, according to the categorical negativity theory and the evolutionary threat hypothesis, negative (vs. neutral and positive) stimuli attract more attention; in particular, stimuli that convey threat signals to survival (e.g., fearful facial expression) during evolution could attract more attention [24,25]. In other words, processing fearful stimuli costs more attention resources, so we hypothesized that fear priming would be detrimental to an individual’s estimation strategy selection adaptivity (hypothesis two). Considering previous studies showing that estimation performances were differently influenced by explicit and implicit fear priming, we test the above-mentioned hypotheses through two experiments, from the perspective of explicit and implicit emotion priming, respectively. Lastly, a previous study showed that participant’s estimation ACC and RT were not influenced by math anxiety level when they were required to complete an estimation strategy selection task [17]. Therefore, we speculated that similar findings would be obtained in the present study and hypothesized that there would be no significant correlation between participants’ estimation performance (ACC and RT) and math anxiety when they were required to complete the estimation task under different snapshot emotional priming conditions (hypothesis three).

## 2. Experiment 1: The Influence of Explicit Emotion Priming on Estimation Strategy Selection

### 2.1. Method

#### 2.1.1. Participants

A previous similar study showed that the 2 (estimation problem type) × 3 (emotion type) experimental design had a significant two-way interaction (effect size $\eta_{P}^{2}$ = 0.26) [18]. A priori power analysis conducted by MorePower 6.0.4 [26] showed that at least 24 participants were required to achieve an effect size $\eta_{P}^{2}$ = 0.26 (α = 0.05, power level = 0.95) when focusing on the interaction between emotion types (happy, fear, and neutral) and estimation strategy types (DU and UD). To ensure that our sample size was large enough, thirty participants were recruited. Three participants were removed due to broken equipment and missing files; twenty-seven (M ± SD, 20.04 ± 0.76 years old, and range from 19–21 years old) right-handed college students were included. All participants had normal or corrected to normal visual acuity. All participants signed an informed consent form before the experiment. All participants received a small amount of money for their participation. The study was conducted according to the guidelines of the Declaration of Helsinki, and approved by the Research Ethics Committee of Yangzhou University.

#### 2.1.2. Materials

(1) Facial expression images

The facial expression images used in the present study were selected from the NimStim Set of Facial Expressions [27]. Before the formal experiment, thirty-two participants were recruited to take part in the expression recognition test. For each facial expression image, participants had to assess the emotion they perceived and the certainty of their answer (on a 9-point scale). Only images with a mean accuracy greater than 0.8, i.e., successfully recognized by 26 participants, and with a median of certainty greater than 8 were adopted. Additionally, twenty-five new participants were recruited to rate the valence and arousal of all images on a 9-point scale. “1” stands for very unpleasant or not at all arousing, “9” stands for very pleasant or very arousing. The above-mentioned participants didn’t participant in the formal experiment. Finally, fifty-four facial expression images of 18 different models (nine females) showing happy, neutral, and fearful expressions were selected. The numbers of the models in the database were as follows: female, 1, 2, 3, 6, 9, 12, 13, 17, 19; men, 20, 21, 24, 27, 33, 36, 37, 41, 42. The results of repeated-measure ANOVA showed that the main effect of valence was significant, F(2,34) = 28.95, *p* < 0.001, $\eta_{P}^{2}$ = 0.630. The valence of happy (M ± SD, 6.23 ± 1.12) was higher than neutral (4.61 ± 0.75, *p* = 0.005) and fear (3.49 ± 0.84, *p* < 0.001), and neutral was higher than fear (*p* < 0.001). Meanwhile, the main effect of arousal was not significant, F(2,34) = 0.49, *p* = 0.617, $\eta_{P}^{2}$ = 0.028. The arousal of the three emotions was as follows: happy, M ± SD, 5.62 ± 0.49; neutral, 5.57 ± 0.50; fear, 5.70 ± 0.45. In all, there was a significant difference between the valences of different emotions while not in arousal, so the influence of the snapshot emotional priming stimuli’s valence (rather than arousal degree) on the execution of estimation strategies can be effectively investigated. Each image was cropped into an ellipse using Photoshop 8.0. The viewing angle was 4.52° (horizontal) × 6.75° (vertical), and the screen resolution was 100 pixels per inch.

(2) Multiplication formulas

One hundred and eight two-digit multiplication formulas were adopted; fifty-four formulas were suitable for DU strategy, while the other fifty-four formulas were suitable for UD strategy. The principles of selecting the multiplication formulas followed previous studies [2,19,22,23]. The details were as follows: (1) no operand had its closest decade equal to 10 (e.g., 13 × 67), or 100 (e.g., 23 × 98); (2) no operand had 0 (e.g., 21 × 70) or 5 as unit digits (e.g., 35 × 67); (3) operands were not repeated in the decade (e.g., 21 × 27) or unit (e.g., 67 × 87); (4) no digits were repeated within operands (e.g., 22 × 37); (5) no tie problems were used (e.g., 38 × 38).The multiplication formulas were displayed on the screen with a black background. The font used was Times New Roman, size 60 pt.

(3) Math anxiety scale

The revised mathematics anxiety rating scale (R-MARS) [17,28] was used to measure participants’ math anxiety levels. The R-MARS consists of 21 items. The total Cronbach’s coefficient α of the questionnaire was 0.932, the split-half reliability was 0.885, and the reliability coefficient was 0.929, which indicate that the R-MARS has good reliability and validity. Participants were asked to complete the R-MARS with the Likert 5-point scale. “1” stands for “not anxious” and “5” points for “very anxious”.

#### 2.1.3. Procedure

**Figure 1:**
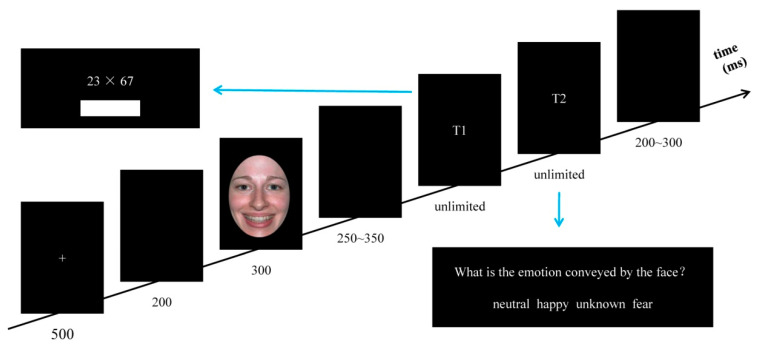
Illustration of one trial in Experiment 1.

The experiment was conducted in a soundproof room. The procedure was presented with E-prime 2.0 (Psychology Software Tools Inc., Pittsburgh, PA, USA). The experimental procedure comprised four blocks, while each block included 54 trials, for a total of 216 trials. As shown in Figure 1, the order of the stimuli presented in a single trial was as follows: each trial started with a white “+” (500 ms), followed by a blank (200 ms), the facial expression image (300 ms), a blank (250~350 ms), the multiplication computational estimation task (MCE task, unlimited, T1), the emotion judgment task (EJ task, unlimited, T2), and then a blank (200~300 ms). Before the formal experiment, all participants were told how to use the DU and UD strategies. They were told freely to complete the estimation task either with DU or UD strategies for each multiplication formula, which needed to be as close to the correct answer as possible. In the MCE task, the multiplication formula was presented in the middle of the screen. As soon as participants produced a solution, they were asked to press the “Q” key and enter their solution; after that, they were required to enter the “enter” key. Otherwise, the computer would not go to the next screen. In the EJ task, participants were required to judge which emotion was conveyed by the facial expression image. Participants were required to press the “D” key with their left middle finger when they judged the emotion expressed by the facial image as neutral, key “F” with their left index finger when judged as happy, key “J” with their right middle finger when they did not know which emotion was conveyed, and key “K” with their right index finger when judged as fear. Participants were required to respond as accurately and quickly as possible in the MCE and EJ tasks.

Pseudo-random design was been adopted in the present study; each experimental condition did not appear continuously more than three times. To ensure that participants fully understood the experiment process, twelve practice trials were provided before the formal experiment. To control for fatigue, all participants were required to rest for 2 min after finishing each block and then carry out the following block. Feedback was provided in the practice phase, while no feedback was provided in the formal phase.

#### 2.1.4. Data Analysis

Only trials in which participants successfully completed the EJ task were included in the analysis. The experimental data were analyzed by analysis of variances (ANOVAs) and t-test. Independent variables were estimation strategy types (DU, UD) and priming emotion types (fear, happy, and neutral), and dependent variables were participants’ ACC and RT when completing the estimation task. In the correctly answered trials, the participants’ response time was the period from when the multiplication formula was presented to when they pressed the “Q” key to enter their answer. The statistical analysis was conducted using SPSS 26.0. The Pearson correlation was adopted to analyze the relationship between estimation performance and math anxiety. The Greenhouse–Geisser correction was applied to the *p* values, while the degree of freedom did not satisfy the spherical test hypothesis. All post-hoc tests included Bonferroni’s correction. Partial eta-squared ($\eta_{P}^{2}$) was used to describe effect sizes.

### 2.2. Results

#### 2.2.1. Strategy Distribution

Strategy distribution is defined as how often or how many times each strategy is used. According to the estimation results of the participants, which strategy the participants have adopted is judged. If participants adopted the DU strategy, the corresponding trial was coded as “Method 1”; if they adopted the UD strategy, the corresponding trial was coded as “Method 2”, while trials in which other strategies were adopted were coded as “Method 3”. “Other strategies” means strategies besides DU and UD (e.g., RD). The sum of the proportions using “Method 1”, “Method 2”, and “Method 3” is 1. The probabilities of using DU, UD, and the other strategies under different priming conditions are shown in Table 1.

The results of the one-sample *t*-test showed that the probability of using DU and UD strategies under fear, happy, and neutral priming conditions were higher (*ps* < 0.001) than the probability level (0.33). In contrast, the probability of using the other strategies under three different snapshot emotional priming conditions were lower than (*ps* < 0.001) than probability level (0.33), which suggests that the participants successfully used the DU and UD strategies and adopting other strategies was a low-probability event. To compare whether there was a difference in the probability of using DU and UD strategies under different snapshot emotional priming conditions, a 2(estimation strategy types: DU, UD) × 3(priming emotion types: fear, happy, and neutral) ANOVA was conducted. The results showed that the main effect of priming emotion types was not significant, F(2,52) = 0.549, *p* = 0.581, $\eta_{P}^{2}$ = 0.021. The main effect of estimation strategy types was not significant, F(1,26) = 0.618, *p* = 0.439, $\eta_{P}^{2}$ = 0.023. The interaction effect of these two factors was not significant, F(2,52) = 0.067, *p* = 0.897, $\eta_{P}^{2}$ = 0.003. These results show that strategy distribution was not influenced by snapshot emotional priming; meanwhile, participants did not show a special preference for DU or UD strategies, which provided the basis for the subsequent analysis of strategy selection accuracy, RT, and strategy selection flexibility.

#### 2.2.2. The ACC and RT When Completing the MCE Task

Only when participants solved the DU problem with DU strategy (e.g., doing 30 × 70 = 2100 for 32 × 67), or solving the UD problem with UD strategy (e.g., doing 40 × 60 = 2400 for 37 × 62) were their responses considered as correct. Otherwise, their answer was judged as wrong. We analyzed the ACC and RT separately using 2 (estimation strategy types: DU, UD) × 3 (priming emotion types: fear, happy, and neutral) repeated-measure ANOVAs. The results were as follows.

**Figure 2:**
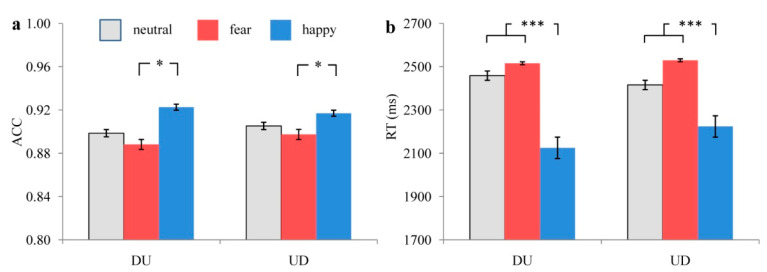
The ACC (**a**) and RT (**b**) when completing the MCE task under different priming conditions. “*” means “*p* < 0.05”, “***” means “*p* < 0.001”. The grey bar means neutral priming condition, the red bar means fear priming condition, and the blue bar means happy priming condition.

In terms of ACC, the main effect of priming emotion types was marginally significant, F(2,52) = 2.882, *p* = 0.065, $\eta_{P}^{2}$ = 0.100. The ACC under happy priming condition was higher (*p* = 0.040) than under fear priming. The main effect of estimation strategy types was not significant, F(1,26) = 0.210, *p* = 0.650, $\eta_{P}^{2}$ = 0.008. The ACCs under DU and UD conditions showed no significant difference (*p* = 0.650). The interaction effect of these two factors was not significant, F(2,52) = 0.312, *p* = 0.662, $\eta_{P}^{2}$ = 0.012. Simple effect analysis showed that both when using DU and UD strategies, the ACCs under three snapshot emotional priming conditions (fear, happy, and neutral) showed no significant difference (*ps* > 0.05). The ACCs when completing the estimation task under different snapshot emotional priming conditions are shown in Figure 2a.

In terms of RT, the main effect of priming emotion types was significant, F(2,52) = 20.100, *p* < 0.001, $\eta_{P}^{2}$ = 0.436. RT under happy priming condition was shorter (*ps* < 0.001) than under fear and neutral conditions, while RT under fear and neutral conditions showed no significant difference (*p* = 0.531). The main effect of estimation strategy types was not significant, F(1,26) = 0.386, *p* = 0.540, $\eta_{P}^{2}$ = 0.015. RTs under DU and UD conditions showed no significant difference (*p* = 0.540). The interaction effect of priming emotion types and estimation strategy types was not significant, F(2,52) = 2.553, *p* = 0.088, $\eta_{P}^{2}$ = 0.089. Simple effect analysis showed that both when using DU and UD strategies, RTs under happy priming conditions were shorter (*ps* < 0.001) than under fear and neutral priming conditions, while RTs under fear and neutral priming conditions showed no significant difference (*ps* > 0.05). RTs when completing the estimation task under different snapshot emotional priming conditions are shown in Figure 2b. Additionally, the Pearson correlation analysis results showed that the correlations between ACC and RT under different conditions were not significant (*ps* > 0.05) (Table 2), which suggested that no RT and response accuracy trade-off was found in the present study.

#### 2.2.3. The Relationship between Estimation Performance and Math Anxiety

To examine the relationship between individuals’ estimation performance (ACC and RT) and math anxiety, Pearson correlation analysis was adopted. The results showed that the correlations between estimation ACC and math anxiety, and estimation RT and math anxiety under different priming conditions were not significant (*ps* > 0.05) (Table 3), which suggests that individuals’ estimation performance was not influenced by math anxiety under explicit snapshot emotional priming condition.

#### 2.2.4. Strategy Selection Adaptivity

According to previous studies [29,30,31], we defined strategy selection adaptivity as follows: for a particular estimation question, participants selected the problem-based strategy that best-balanced proximity to the correct answer. If the problem-based strategy was adopted, then the participant’s strategy selection on this problem was considered adaptive, while the opposite was considered maladaptive. For example, when estimating the result of 32 × 67, using the DU strategy (doing 30 × 70 = 2100 for 32 × 67) can obtain the most accurate result; if participants adopted the DU strategy, then their strategy selection was judged as adaptive. In the present experiment, we adopted the ACC and RT that met this criterion to measure the adaptivity of strategy selection. The ACC-related results showed that the main effect of priming emotion types was marginally significant, F(2,52) = 2.760, *p* = 0.073, $\eta_{P}^{2}$ = 0.096. Post-hoc pair-wise comparison results showed that the ACC-related strategy selection adaptivity was higher (*p* = 0.047) under happy priming condition than under fear priming condition. The RT-related results showed that the main effect of priming emotion types was significant, F(2,52) = 20.100, *p* < 0.001, $\eta_{P}^{2}$ = 0.436. Post-hoc pair-wise comparison results showed that RT-related strategy selection adaptivity was higher (*ps* < 0.001) under happy priming condition than under fear and neutral priming condition, while RT-related strategy selection adaptivity under fear and neutral priming condition showed no significant difference (*p* = 0.531). These results showed that the participants’ strategy selection adaptivity was influenced by explicit snapshot emotional priming.

Additionally, to examine the relationship between individuals’ strategy selection adaptivity and math anxiety, the Pearson correlation analysis was adopted. The results show that the correlations between individuals’ strategy selection adaptivity under three priming conditions and math anxiety were not significant (*ps* > 0.05). The correlations under different priming conditions were as follows: fear, r = −0.087, *p* = 0.668; happy, r = −0.149, *p* = 0.457; neutral, r = −0.110, *p* = 0.584. These results suggest that individuals’ strategy selection adaptivity was not influenced by math anxiety under explicit snapshot emotional priming condition.

In summary, Experiment 1 showed that strategy distribution was not influenced by explicit snapshot emotional priming. Meanwhile, estimation performance was affected by explicit snapshot emotional priming. Firstly, happy priming contributed to improving individuals’ estimation performance. To be more specific, participants’ RT when completing the estimation task was shorter under the happy priming condition than under neutral and fear priming conditions, and their ACC when completing the estimation task was higher under the happy priming condition, which suggests that compared with neural and fear, the happy priming effect was better. Secondly, fear priming did not adversely affect individuals’ estimation performance. Under fear and neutral priming conditions, the corresponding ACC and RT when completing the estimation task showed no significant difference, which suggests that the influence of fear and neutral priming on individuals’ estimation performance had no significant difference, i.e., individuals’ estimation performance was equally influenced by fear and neutral priming. Thirdly, individuals’ ACC and RT were not affected by their math anxiety under explicit snapshot emotional priming conditions. Furthermore, individuals’ strategy selection adaptivity under different explicit snapshot emotional priming conditions was not affected by math anxiety. In Experiment 2, we explored whether such effects of happy and fear priming would be observed under implicit snapshot emotional priming conditions.

## 3. Experiment 2: The Influence of Implicit Emotion Priming on Estimation Strategy Selection

### 3.1. Method

#### 3.1.1. Participants

Like Experiment 1, thirty right-handed college students (M ± SD, 20.03 ± 0.81 years old, and ranging from 19–21 years old) were recruited. All participants had normal or corrected to normal visual acuity. All participants signed an informed consent form before the experiment. All participants received a small amount of money for their participation. The study was conducted according to the guidelines of the Declaration of Helsinki, and approved by the Research Ethics Committee of Yangzhou University.

#### 3.1.2. Materials

The materials used in Experiment 2 were the same as those used in Experiment 1.

#### 3.1.3. Procedure

**Figure 3:**
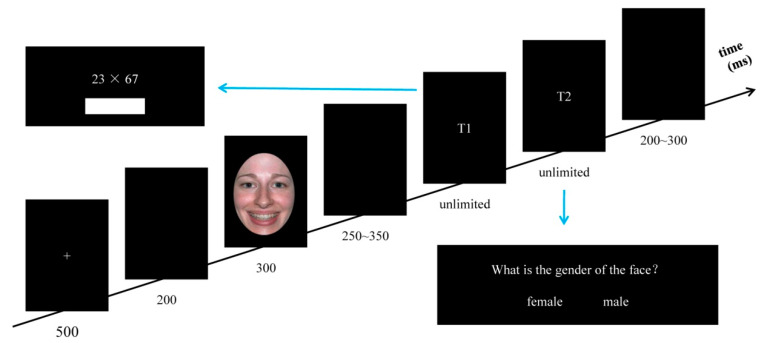
Illustration of one trial in Experiment 2.

The experimental procedure was the same as in Experiment 1, except that the emotion judgment task in Experiment 1 was replaced by the gender judgment (GJ) task. A large number of previous studies indicated that judging the gender of a face could induce individuals’ implicit emotional experience when emotional facial expression images used as stimuli [32,33]. Therefore, the gender judgment task was adopted to achieve the goal of implicit snapshot emotional priming. In the GJ tasks, participants were required to press the ‘‘F’’ key with their left middle finger if the correct answer was presented on the left side, and press the “J” key with their right finger if the correct answer was presented on the right side. The sequence of stimulus presentation in a single trial is shown in Figure 3.

#### 3.1.4. Data analysis

The data analysis methods used in Experiment 2 were the same as those used in Experiment 1.

### 3.2. Results

#### 3.2.1. Strategy Distribution

Similar to Experiment 1, if participants adopted the DU strategy, the corresponding trial was coded as “Method 1”; if they adopted the UD strategy, the corresponding trial was coded as “Method 2”, while trials in which other strategies were adopted were coded as “Method 3”. The sum of the proportions using “Method 1”, “Method 2”, and “Method 3” is 1. The probabilities of using DU, UD, and other strategies under different priming conditions are shown in Table 4.

The results of the one-sample t-test showed that the probability of using DU and UD strategies under fear, happy, and neutral priming conditions were higher (*ps* < 0.001) than the probability level (0.33), while the probability of using the other strategy under three different snapshot emotional priming conditions were lower than (*ps* < 0.001) than probability level (0.33), which suggested that the participants successfully used the DU and UD strategies and adoption of other strategies was a low-probability event. To compare whether there was a difference in the probability of using DU and UD strategies under different snapshot emotional priming conditions, a 2(estimation strategy types: DU, UD) × 3(priming emotion types: fear, happy, and neutral) ANOVA was conducted. The results show that the main effect of priming emotion types was not significant, F(2,58) = 0.488, *p* = 0.616, $\eta_{P}^{2}$ = 0.017. The main effect of estimation strategy types was not significant, F(1,29) = 0.382, *p* = 0.541, $\eta_{P}^{2}$ = 0.013. The interaction effect of these two factors was not significant, F(2,58) = 2.188, *p* = 0.121, $\eta_{P}^{2}$ = 0.070. These results show that strategy distribution was not influenced by implicit snapshot emotional priming; meanwhile, participants did not show a special preference for DU or UD strategy, which provided the basis for the subsequent analysis of strategy selection accuracy, RT, and strategy selection flexibility.

#### 3.2.2. The ACC and RT When Completing the MCE task

Similar to Experiment 1, we analyzed the ACC and RT separately by using 2 (estimation strategy types: DU, UD) × 3 (priming emotion types: fear, happy, and neutral) repeated-measures ANOVAs. The results were as follows.

**Figure 4:**
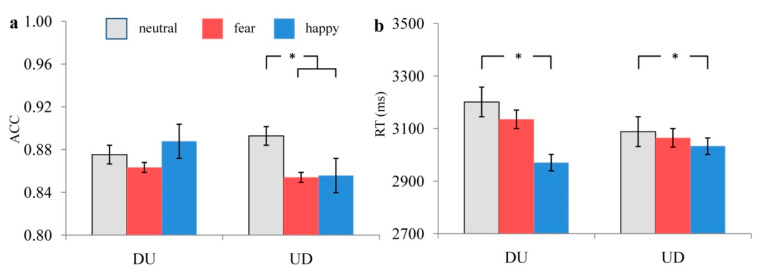
The ACC (**a**) and RT (**b**) when completing the MCE task under different priming conditions. “*” means “*p* < 0.05”. The grey bar means neutral priming condition, the red bar means fear priming condition, and the blue bar means happy priming condition.

In terms of ACC, the interaction effect of these two factors was significant, F(2,58) = 3.328, *p* = 0.043, $\eta_{P}^{2}$ = 0.103. Simple effect analysis showed that when using the UD strategy, the ACC under the neutral priming condition was higher than under implicit fear (*p* = 0.013) and implicit happy (*p* = 0.026) conditions, while the ACC under the latter two conditions showed no significant difference (*p* = 0.887). Meanwhile, when using the DU strategy, the ACCs under three snapshot emotional priming conditions (fear, happy, and neutral) showed no significant difference (*ps* > 0.05). The main effect of priming emotion types was not significant, F(2,58) = 2.406, *p* = 0.099, $\eta_{P}^{2}$ = 0.077.The ACCs under three snapshot emotional priming conditions (fear, happy, and neutral) showed no significant difference(*ps* > 0.05). The main effect of estimation strategy types was not significant, F(1,29) = 0.890, *p* = 0.353, $\eta_{P}^{2}$ = 0.030. The ACCs under DU and UD conditions showed no significant difference (*p* = 0.353). The ACCs when completing the estimation task under different snapshot emotional priming conditions are shown in Figure 4a.

In terms of RT, the main effect of priming emotion types was significant, F(2,58) = 4.433, *p* = 0.016, $\eta_{P}^{2}$ = 0.133. RT under the implicit happy priming condition wasshorter (*p* = 0.012) than under neutral conditions. Meanwhile, RT between the implicit happy and fear priming conditions showed no significant difference (*p* > 0.05). The main effect of the estimation strategy types was not significant, F(1,29) = 0.699, *p* = 0. 410, $\eta_{P}^{2}$ = 0.024. RTs under DU and UD conditions showed no significant difference (*p* = 0.410). The interaction effect of priming emotion types and estimation strategy types was not significant, F(2,58) = 1.913, *p* = 0.172, $\eta_{P}^{2}$ = 0.062. Simple effect analysis showed that when using the DU strategy, RT under the implicit happy priming condition was shorter than under neutral conditions (*p* = 0.007), while RTs under the other conditions showed no significant difference (*ps* > 0.05). Meanwhile, when using the UD strategy, RTs under three snapshot emotional priming conditions (fear, happy, and neutral) showed no significant difference (*ps* > 0.05). RTs when completing the estimation task under different snapshot emotional priming conditions are shown in Figure 4b. Additionally, the Pearson correlation analysis results showed that the correlations between ACC and RT under different conditions were not significant (*ps* > 0.05) (Table 5), which suggested that no RT and response accuracy trade-off was found in the present study.

#### 3.2.3. The Relationship between Estimation Performance and Math Anxiety

The Pearson correlation analysis results showed that the correlations between estimation ACC and math anxiety, and estimation RT and math anxiety under different implicit priming conditions were not significant (*ps* > 0.05) (Table 6), which suggests that individuals’ estimation performance was not influenced by math anxiety under implicit snapshot emotional priming conditions.

#### 3.2.4. Strategy Selection Adaptivity

Similar to Experiment 1, we adopted the ACC and RT that met this criterion to measure the adaptivity of strategy selection. The ACC-related results showed that the main effect of priming emotion types was not significant, F(2,58) = 2.760, *p* = 0.122, $\eta_{P}^{2}$ = 0.070. The RT-related results showed that the main effect of priming emotion types was significant, F(2,58) = 4.432, *p* = 0.016, $\eta_{P}^{2}$ = 0.133. Post-hoc pair-wise comparison results showed that the RT-related strategy selection adaptivity was higher (*p* = 0.012) under the implicit happy priming condition than under neutral priming condition, while the RT-related strategy selection adaptivity under fear and neutral priming conditions showed no significant difference (*p* = 0.217). These results showed that the participants’ strategy selection adaptivity was influenced by implicit snapshot emotional priming. Under implicit snapshot emotional priming conditions, RT can better reflect the individual’s strategy selection adaptivity than ACC.

The Pearson correlation analysis results showed that the correlations between individuals’ strategy selection adaptivity under three priming conditions and math anxiety were not significant (*ps* > 0.05). The correlations under different priming conditions were as follows: fear, r = −0.194, *p* = 0.305; happy, r = −0.153, *p* = 0.418; neutral, r = −0.137, *p* = 0.469. These results suggested that individuals’ strategy selection adaptivity was not influenced by math anxiety under implicit snapshot emotional priming conditions.

In summary, Experiment 2 showed that strategy distribution was not influenced by implicit snapshot emotional priming. Meanwhile, estimation performance was affected by implicit snapshot emotional priming. Firstly, compared with neutral, implicit happy priming reduced individuals’ RT when using both DU and UD strategies, with reduced ACC when using UD strategy. Secondly, implicit fear (vs. neutral) priming was detrimental to individuals’ estimation performance, i.e., implicit fear priming reduced individuals’ estimated ACC when using the UD strategy. Thirdly, both individuals’ estimation ACC and RT were not influenced by their math anxiety under implicit snapshot emotional priming conditions. Meanwhile, individuals’ strategy selection adaptivity under different implicit snapshot emotional priming conditions was not influenced by math anxiety.

## 4. Discussion

The goal of the present study was to explore the influence of snapshot emotional priming (explicit and implicit) on estimation strategy selection for the first time. Participants were required to complete the MCE task, which was preceded by an emotional facial expression. Then, they were required to complete the EJ task (Experiment 1, explicit snapshot emotional priming) or the GJ task (Experiment 2, implicit snapshot emotional priming). In Experiment 1 and Experiment 2, effects of snapshot emotional priming on participants’ estimation performance were observed while completing the MCE task. Under explicit snapshot emotional priming conditions, individuals’ strategy selection adaptivity was positively influenced by happy priming, while not influenced by fear priming. Under implicit snapshot emotional priming conditions, the influence of happy priming on individuals’ strategy selection adaptivity was complex: happy priming reduced the individuals’ estimation ACC when using UD strategy, but improved the individual’s RT. Meanwhile, individuals’ strategy selection adaptivity was negatively influenced by fear priming. Both in Experiment 1 and Experiment 2, strategy distribution was not affected by snapshot emotional priming.

### 4.1. The Influence of Happy Priming on Estimation Strategy Selection

Under explicit priming conditions (Experiment 1), compared with neutral priming, happy priming reduced individuals’ RT, while their corresponding ACC showed no significant difference; compared with fear; under happy priming condition, participants’ ACC was higher and their RT was shorter. These findings indicate that participants’ strategy selection adaptivity was the best under happy priming conditions, which was consistent with the broaden-and-build theory of positive emotions [20,21,34]. The broaden-and-build theory of positive emotions posits that compared with neural and negative emotional experiences, positive emotional experience contributes to expanding attention span and cognitive categorization, and it aids individuals to perform task switching, thus improving individuals’ cognitive flexibility [21,35,36].

Additionally, under implicit priming conditions (Experiment 2), the influence of implicit happy priming on individuals’ estimation performance when using DU and UD strategies was complicated. For the RT indicator, implicit happy priming reduced individuals’ RT when using both DU and UD strategies. For the ACC indicator, individuals’ estimation ACC was not influenced by implicit happy priming when using the DU strategy. However, individuals’ estimation ACC was reduced by implicit happy priming when using the UD strategy. Therefore, the DU strategy-related results of Experiment 2 are supported by the broaden-and-build theory of positive emotions, and the RT (vs. ACC) when using the UD strategy could be explained by the above-mentioned theory. Generally speaking, the positive effect of happy priming on estimation strategy selection outweighed the adverse impact. Hypothesis one was partly confirmed.

### 4.2. The Influence of Fear Priming on Estimation Strategy Selection

In Experiment 1 (explicit priming), under fear and neural priming conditions, individuals’ estimation ACC and RT showed no significant difference, which was inconsistent with our hypothesis. In a recent study [13], participants were asked to complete the two-digit multiplication estimation task under neutral or negative priming conditions by freely adopting RD or RU strategy. The results showed that participants’ estimation ACC showed no significant difference under neutral and negative priming conditions, which supported our findings. However, Lallement and Lemaire (2021) found that participants’ RT was longer under negative (vs. neutral) priming conditions, which was inconsistent with our findings. This discrepancy may be due to the different presentation times of the priming stimuli that were adopted, since a previous study showed that short-term stress boosted individuals’ cognitive performance while long-term stress impaired cognitive performance [37].

In Experiment 2 (implicit priming), participants’ estimation RTs were not influenced by implicit fear priming when they adopted the DU and UD strategies. Meanwhile, their estimation ACC was lower under implicit fear (vs. neutral) priming when they adopted the UD (but not DU) strategy. The results of Experiment 2 showed that implicit fear priming was detrimental to individuals’ estimation performance when using the UD strategy. Hypothesis two was partly confirmed. Additionally, previous studies have explored the influence of both explicit and implicit fear priming on estimation strategy execution [2,19], but only the DU strategy has been adopted. These studies did not examine the influence of fear priming on the execution of the UD strategy. The present study showed that individuals’ estimation performances when using DU and UD strategies were affected differently by implicit fear priming, which reflects the necessity of examining the influence of snapshot emotional priming on individuals’ choice of multiple (not only one) estimation strategies.

### 4.3. The Influence of Math Anxiety on Estimation Performance

Both Experiment 1 and Experiment 2 showed that the correlations between participants’ estimation ACC and math anxiety, and RT and math anxiety were not significant, which suggests that participants’ estimation performance was not influenced by their math anxiety level, both under explicit and implicit snapshot emotional priming conditions. The above-mentioned findings are supported by a previous study [17]. Meanwhile, individuals’ strategy selection adaptivity under snapshot emotional priming conditions (explicit and implicit) was not influenced by math anxiety, which is consistent with a previous study [5]. However, another study [38] showed that estimation strategy selection was influenced by math anxiety. In detail, retrieval strategy was used less frequently in high-anxious adults than in low-anxious adults. In addition, another study found that high (vs. low) math anxiety individuals performed worse in complex (but not simple) arithmetic tasks [39], which is inconsistent with our findings. This discrepancy may be due to the difficulty of the estimation task, i.e., the participants in the present study were college students and the MCE task may be too simple for them. Future studies can increase the difficulty of the estimation task to confirm this speculation. Hypothesis three was confirmed.

Additionally, we obtained an interesting result; participants’ ACC was reduced when the UD (but not DU) strategy was used in Experiment 2, which suggests that individuals were more susceptible to implicit emotional experience when they chose to use the UD strategy. Combined with the RT-related results, it can be seen that participants chose the UD strategy faster under implicit happy (vs. neutral) priming condition, but this was at the expense of ACC. Implicit fear (vs. neutral) priming did not reduce individuals’ estimation speed when choosing the UD strategy, but their corresponding ACC was reduced, which suggests that implicit fear priming was detrimental to choosing UD strategy. Additionally, a recent study [40] showed that DU is harder than UD. Therefore, the present study may indicate that simple strategies are more susceptible to implicit fear priming; future studies can verify this hypothesis.

## 5. Conclusions

The present study found that estimation strategy distribution was not affected by snapshot emotional priming (explicit and implicit). Under explicit priming conditions, happy priming contributed to improving individuals’ estimation performance (shorter RT), while fear priming showed no significant influence on individuals’ estimation performance. Under implicit priming conditions, happy and fear priming reduced individuals’ estimation ACC when using the UD strategy, but happy priming improved individuals’ RT when using DU and UD strategies. Both under explicit and implicit priming conditions, individuals’ estimation ACC, RT, and strategy selection adaptivity were not influenced by their math anxiety level. Both under explicit and implicit priming conditions compared with neutral, individuals’ strategy selection adaptivity was positively influenced by happy priming, while not affected by fear priming. The present study indicated that happy priming was not always beneficial to individuals’ estimation performance, and fear priming was not always detrimental to individual’s estimation performance.

These findings contribute to expanding our understanding of the relationship between snapshot emotional priming and estimation strategy selection to a certain extent, but there are still some limitations. Firstly, it is unclear whether the findings of this study can be applied to other age groups, e.g., children. Previous studies have shown that estimation strategy selection is significantly influenced by age, with older individuals’ estimation strategy selection performance being better than younger ones; the older individuals selected a variety of problem-based strategies flexibly, while the younger tended to use fewer strategies [4,11]. Future studies could verify whether the findings of the present study apply to children. Secondly, the relationship between math anxiety and estimation performance needs to be viewed with caution. The present study found that the correlation between individuals’ estimation ACC/RT and math anxiety level was not significant, which is consistent with a previous study [17]. However, Si et al. (2014) found that high (vs. low) math anxiety individuals’ N100 and N400 amplitudes were larger when completing the estimation strategy selection task, which suggests that behavioral experiments alone are not sufficient to fully reveal the influence of math anxiety on estimation strategy selection. Future research can adopt electrophysiological techniques, such as ERP, fMRI, etc., to deeply study the relationship between math anxiety and estimation strategy selection from the perspective of physiological mechanisms. Lastly, the gender judgment task may be biased by the participant’s gender and sexual orientation; this idea can be tested by comparing the influence of different implicit emotion processing tasks on estimation strategy selection (e.g., gender judgment task vs. age judgment task) in future studies.

## Figures and Tables

**Table 1:** The probability of using different strategies under different conditions in Experiment 1 (M ± SD).

	Fear	Happy	Neutral
DU	0.482 ± 0.034 ***	0.485 ± 0.034 ***	0.484 ± 0.037 ***
UD	0.488 ± 0.040 ***	0.490 ± 0.023 ***	0.486 ± 0.042 ***
Other strategy	0.030 ± 0.057 ***	0.025 ± 0.051 ***	0.030 ± 0.070 ***

Note: “***” means “*p* < 0.001”.

**Table 2:** The Pearson correlation between ACC and RT under different conditions.

	Negative	Positive	Neutral
DU (*p*)	−0.264 (0.183)	0.274 (0.166)	0.105 (0.604)
UD (*p*)	0.254 (0.202)	0.099 (0.624)	0.016 (0.936)

**Table 3:** The Pearson correlation between estimation performance and math anxiety.

		Negative	Positive	Neutral
ACC	DU (*p*)	−0.265 (0.181)	0.121 (0.547)	−0. 095 (0.637)
	UD (*p*)	0.067 (0.738)	−0.178 (0.374)	−0.024 (0.905)
RT	DU (*p*)	−0.080 (0.693)	−0.177 (0.377)	−0.072 (0.721)
	UD (*p*)	−0.092 (0.648)	−0.116 (0.566)	−0.150 (0.456)

**Table 4:** The probability of using different strategies under different conditions in Experiment 2 (M ± SD).

	Fear	Happy	Neutral
DU	0.474 ± 0.037 ***	0.478 ± 0.033 ***	0.473 ± 0.048 ***
UD	0.484 ± 0.037 ***	0.471 ± 0.046 ***	0.480 ± 0.039 ***
Other strategy	0.042 ± 0.059 ***	0.051 ± 0.073 ***	0.047 ± 0.073 ***

Note: “***” means “*p* < 0.001”.

**Table 5:** The Pearson correlation between ACC and RT under different conditions.

	Negative	Positive	Neutral
DU (*p*)	−0.116 (0.541)	0.037 (0.848)	<0.001 (0.999)
UD (*p*)	0.071 (0.710)	−0.167 (0.378)	−0.291 (0.118)

**Table 6:** The Pearson correlation between estimation performance and math anxiety.

		Negative	Positive	Neutral
ACC	DU (*p*)	0.040 (0.835)	−0.051 (0.789)	0. 179 (0.344)
	UD (*p*)	0.083 (0.663)	0.032 (0.865)	0.042 (0.825)
RT	DU (*p*)	−0.185 (0.329)	−0.153 (0.421)	−0.059 (0.759)
	UD (*p*)	−0.199 (0.291)	−0.149 (0.431)	−0.210 (0.265)

## Data Availability

Data are available, upon reasonable request, by emailing: psyclzhu@yzu.edu.cn.
